# The role of the NLRP3 inflammasome in hypertension-related chronic heart failure and its potential therapeutic targets

**DOI:** 10.3389/fimmu.2026.1780542

**Published:** 2026-07-01

**Authors:** Feilong Sun, Xinyu Zhao, Chunlin Chen, Xue Ma, Fang Liu

**Affiliations:** 1 Department of Cardiology, Xinhua Hospital Affiliated to Dalian University, Dalian, China; 2 Department of Cardiology, Zhongshan Hospital Affiliated to Dalian University, Dalian, China

**Keywords:** cardiac remodeling, chronic heart failure, extracellular ATP, NLRP3 inflammasome, P2X7 receptor, pressure overload

## Abstract

**Background:**

Hypertension-induced pressure overload is a major driver of chronic heart failure (CHF) and is accompanied by persistent sterile inflammation. However, the upstream mechanisms linking mechanical stress to inflammasome activation across disease stages remain insufficiently defined.

**Objective:**

To characterize the stage-specific role of the extracellular ATP–P2X7–NLRP3 inflammasome axis in pressure overload–induced CHF and to assess its therapeutic potential.

**Methods:**

A transverse aortic constriction (TAC) mouse model was established and followed longitudinally using hemodynamic assessment and echocardiography. Extracellular ATP signaling, P2X7 activation, NLRP3 inflammasome priming and assembly, downstream effector responses, histologic remodeling, and inflammatory cell infiltration were evaluated across disease stages. Causality was tested using pharmacologic inhibition (P2X7 antagonism and the NLRP3 inhibitor MCC950) and genetic deletion of Nlrp3.

**Results:**

Pressure overload induced stage-dependent amplification of extracellular ATP–P2X7 signaling, accompanied by progressive activation of NLRP3 inflammasome pathways. These changes were associated with macrophage accumulation, worsening fibrosis, and declining cardiac function. Pharmacologic or genetic disruption of the ATP–P2X7–NLRP3 axis attenuated inflammasome signaling, reduced adverse remodeling, and improved cardiac structure and function.

**Conclusions:**

Extracellular ATP–P2X7 signaling is an upstream driver of NLRP3 inflammasome activation during pressure overload–induced CHF. The ATP–P2X7–NLRP3 axis may therefore represent a stage-informed and therapeutically actionable target for hypertension-related CHF.

## 1 Introduction

Hypertension is a major modifiable risk factor for chronic congestive heart failure (CHF). Sustained pressure overload, together with persistent neurohumoral activation, promotes myocardial hypertrophy and interstitial fibrosis, disrupts microvascular function and energy metabolism, and gradually drives the transition from compensation to overt structural and functional failure (1, 2). Current management therefore emphasizes early and sustained blood-pressure control combined with guideline-directed heart failure therapy to reduce hospitalization and mortality (3). In clinical practice, risk stratification, combination treatment, and timely intensification are used to delay remodeling during potentially reversible stages. Even so, heart failure is now recognized as a systemic and progressive syndrome with multiorgan involvement and substantial residual risk despite contemporary treatment, indicating that mechanisms beyond hemodynamic load remain clinically important.

Despite these advances, many patients continue to experience recurrent symptoms, ongoing structural remodeling, and marked prognostic divergence under standardized blood-pressure control and optimized baseline therapy (4, 5). This pattern suggests that pressure burden alone does not fully explain progression in hypertension-related CHF. Additional pathological processes likely interact with hemodynamic stress to shape disease trajectory and treatment response.

Among these processes, immune and inflammatory activation has emerged as a key mechanistic bridge between hypertensive load and heart failure development. Heart failure, defined by structural and/or functional abnormalities that impair ventricular filling or ejection, is commonly accompanied by a chronic low-grade inflammatory state. Elevated inflammatory mediators are consistently associated with worse cardiac performance, more advanced remodeling, and poorer outcomes (6). However, these observations are largely associative and do not identify which upstream inflammatory regulators act as causal drivers in hypertension-related CHF or which are most suitable for therapeutic targeting.

Clinical heterogeneity further complicates this problem. Hypertension-associated heart failure frequently coexists with metabolic dysfunction, renal impairment, and obesity (7). In patients exposed to sustained pressure overload, the predominant phenotype is often heart failure with preserved ejection fraction (HFpEF), characterized by concentric remodeling, impaired diastolic relaxation, and a substantial comorbidity burden. This heterogeneity suggests that inflammatory intensity and remodeling trajectories vary across patient subsets, which may partly explain why hemodynamic control alone fails to prevent progression in many cases. A central translational challenge, therefore, is to identify inflammatory pathways that do more than track disease severity and instead function as mechanistic drivers of remodeling (8).

Anti-inflammatory therapies have shown benefit in selected cardiovascular settings, but their role in heart failure remains unsettled because clinical efficacy has been inconsistent and patient selection has often lacked mechanistic stratification (9, 10). Targeting single cytokines may be insufficient in heart failure, where multiple sterile danger signals converge and amplify inflammation over time. In addition, inflammatory programs are likely stage-dependent, making intervention timing a critical issue. In hypertension-related CHF, inflammation also interacts with oxidative stress, mitochondrial dysfunction, disturbed ion homeostasis, and sustained neurohumoral signaling (11). Strategies aimed only at downstream inflammatory mediators may therefore leave upstream drivers intact and fail to alter disease progression meaningfully.

Within this framework, the NLRP3 inflammasome has become an important candidate mechanism because it integrates diverse innate immune danger signals into a common inflammatory cascade (12). NLRP3 activation can couple hypertensive stress–related danger signaling to caspase-1 activation, IL-1 family cytokine maturation and release, and inflammatory cell death, thereby providing a plausible mechanistic route from sterile inflammation to fibrosis and cardiac dysfunction (13, 14). Compared with approaches that target individual cytokines, modulation of NLRP3 may offer a more mechanism-oriented strategy to interrupt convergent inflammatory inputs in hypertension-related CHF.

The upstream triggers that engage NLRP3 during pressure overload, however, remain insufficiently resolved across disease stages. Extracellular ATP signaling through the purinergic receptor P2X7 is a strong candidate pathway because it links mechanical stress and cellular injury to inflammasome activation and sterile inflammation. Yet the stage-specific behavior of the ATP–P2X7–NLRP3 axis, its causal contribution to remodeling, and its therapeutic tractability in pressure overload–induced CHF require clearer definition.

Accordingly, this study uses a stage-informed framework to map inflammatory signaling during the progression of pressure overload–driven CHF and to test a hierarchical intervention strategy. Specifically, we aim to (i) define when and how the ATP–P2X7–NLRP3 axis is activated during disease progression, (ii) determine whether pharmacologic and genetic disruption of this pathway causally attenuates inflammatory remodeling, and (iii) evaluate peripheral blood indicators with translational relevance. By linking disease stage, mechanistic signaling, and interventional evidence, this study seeks to provide a clearer rationale for anti-inflammatory targeting in hypertension-related CHF.

## 2 Methods

### 2.1 Study overview and experimental timeline

This study used a transverse aortic constriction (TAC) model to induce sustained pressure overload and to characterize stage-specific activation of the extracellular ATP–P2X7–NLRP3 inflammasome axis during progression to chronic heart failure (CHF). Mice underwent baseline assessment at week 0, followed by sham or TAC surgery and a 12-week observation period. Systolic blood pressure and echocardiography were measured weekly to enable longitudinal tracking of structural and functional cardiac changes.

**Figure 1:**
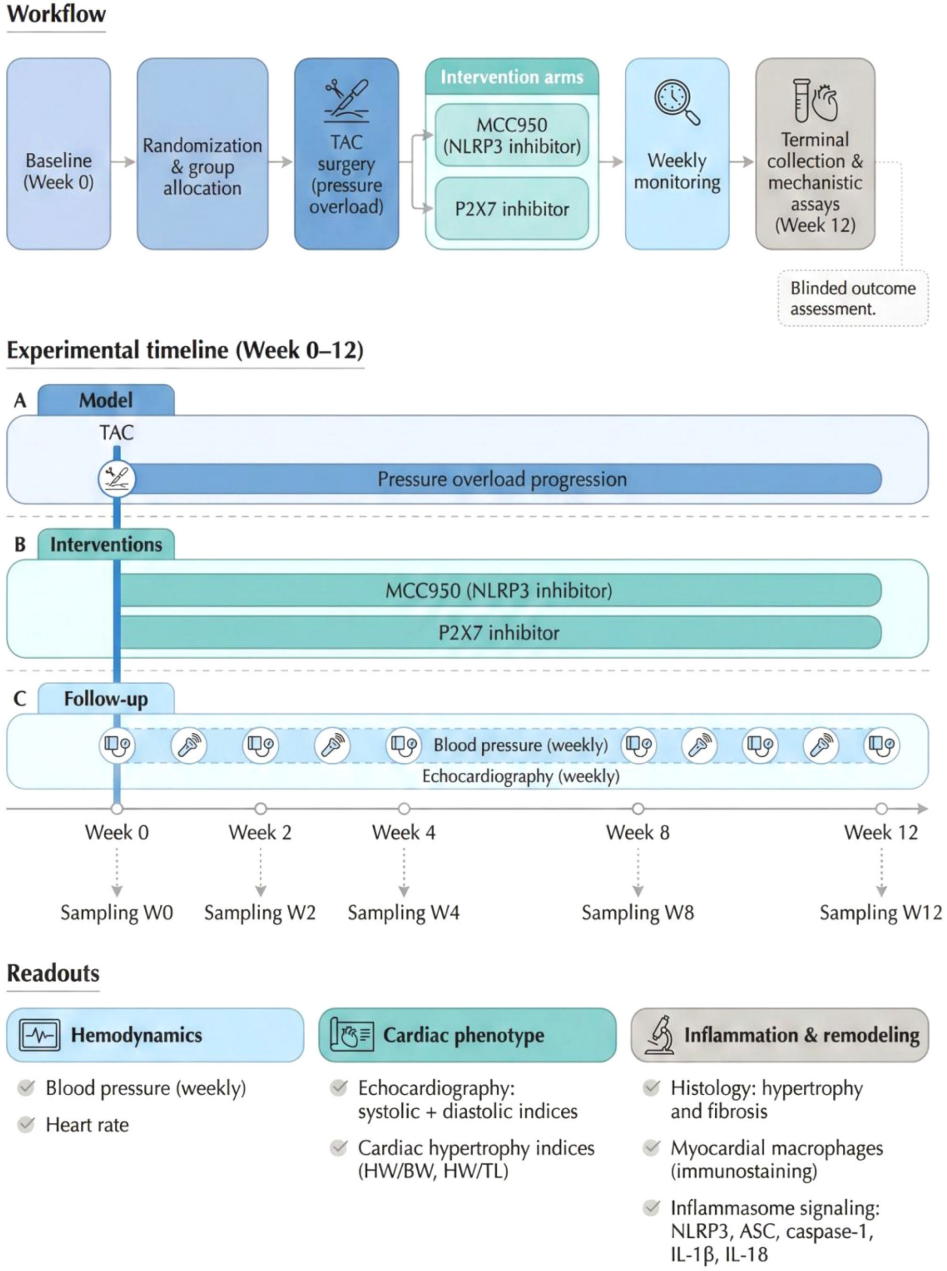
Study workflow and experimental timeline for pressure overload–induced chronic heart failure (CHF) with NLRP3-targeted interventions. **(A)** TAC model establishment and pressure overload progression during the 12-week observation period. **(B)** Pharmacologic intervention schedule with MCC950 or P2X7 inhibitor from week 0 to week 12. **(C)** Longitudinal follow-up scheme, including weekly blood pressure monitoring, echocardiography, and scheduled sampling at weeks 0, 2, 4, 8, and 12.

Terminal sampling was prespecified at weeks 2, 4, 8, and 12, and baseline blood was collected before surgery (week 0). At each terminal time point, left ventricular (LV) tissue was harvested for histological and molecular analyses, and peripheral blood was collected for systemic inflammatory profiling and exploratory translational analyses. The overall workflow and sampling framework are shown in **Figure 1**. Detailed group allocation, intervention windows, and stage-wise sample sizes are provided in **Supplementary Table 1**.

### 2.2 Animals, ethics, randomization, and blinding

All animal procedures were approved by the Institutional Animal Care and Use Committee (IACUC) of Xinhua Hospital, affiliated with Dalian University (approval no. IACUC-2025-147-01), and were performed in accordance with the National Institutes of Health Guide for the Care and Use of Laboratory Animals. Wild-type (WT) C57BL/6J mice (male, 8–10 weeks old, 22–28 g) were used for the pharmacologic intervention arms. Nlrp3−/− mice on a C57BL/6J background (male, 8–10 weeks old) were used for genetic validation. Animals were housed under specific pathogen-free conditions (22 ± 2 °C; 50–60% humidity; 12-h light/dark cycle) with ad libitum access to standard chow and water.

After baseline measurements, animals were assigned to groups using a computer-generated randomization sequence (block size = 4) prepared by an investigator not involved in outcome assessment. Allocation was concealed from investigators performing echocardiography, histologic quantification, flow cytometry, and molecular assays. These assessors remained blinded until completion of statistical analysis.

Prespecified exclusion criteria included perioperative death, severe postoperative complications, or failure to establish effective pressure overload. Failure of model establishment was defined as a transverse aortic constriction (TAC) Doppler peak velocity < 3.5 m/s on postoperative day 3–5 and/or absence of an LV hypertrophic response by echocardiography at week 2. No additional exclusions were applied.

### 2.3 TAC model establishment and perioperative care

TAC surgery was performed under sterile conditions. Mice were anesthetized with isoflurane (3% for induction; 1.5–2.0% for maintenance in 100% oxygen) and placed on a temperature-controlled heating pad (37 °C). Following endotracheal intubation, mice were mechanically ventilated (120 breaths/min; tidal volume 0.2 mL) using a small-animal ventilator. A left thoracotomy was performed at the second intercostal space to expose the transverse aorta. A 27-gauge needle was placed adjacent to the aorta, and a 7–0 silk suture was tied around both the aorta and needle. The needle was then removed to create a standardized constriction. Sham-operated mice underwent the same procedure without ligation. The chest was closed in layers, and negative intrathoracic pressure was restored.

Postoperative analgesia consisted of buprenorphine (0.1 mg/kg, subcutaneous, every 12 h for 48 h) and meloxicam (5 mg/kg, subcutaneous, once daily for 2 days). Mice were monitored daily for body weight, grooming behavior, respiratory status, and wound integrity. Animals showing severe distress or >15% body weight loss were euthanized according to predefined humane endpoints and institutional guidelines.

### 2.4 Pharmacologic and genetic interventions

**Figure 2:**
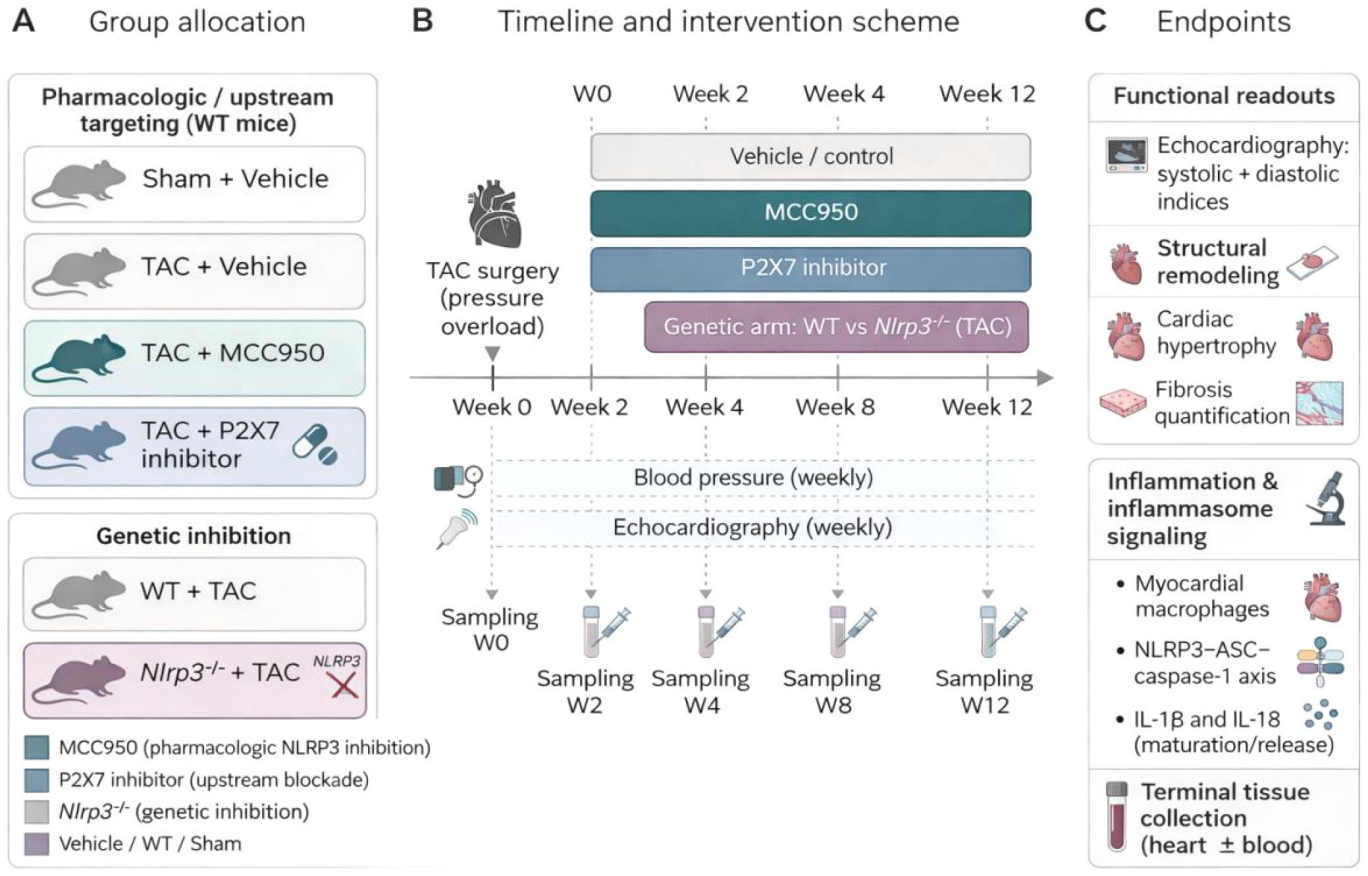
Experimental design for pharmacologic and genetic inhibition of the ATP–P2X7–NLRP3 axis during pressure overload–driven chronic heart failure (CHF). **(A)** Group allocation for pharmacologic and genetic intervention arms, including sham, TAC + vehicle, TAC + MCC950, TAC + P2X7 inhibitor, WT + TAC, and Nlrp3^−/−^ + TAC groups. **(B)** Timeline and intervention scheme showing TAC surgery, treatment administration, weekly blood pressure and echocardiographic monitoring, and scheduled sampling from week 0 to week 12. **(C)** Prespecified endpoints, including functional readouts, structural remodeling, cardiac hypertrophy, fibrosis quantification, inflammation and inflammasome signaling, and terminal tissue collection.

To test pathway hierarchy and causality, both pharmacologic inhibition and genetic disruption of NLRP3 were used. Pharmacologic interventions included (i) MCC950 to inhibit NLRP3 activation and (ii) the selective P2X7 receptor antagonist A-740003 to block upstream ATP–P2X7 signaling (**Figure 2A**). Unless otherwise stated, treatments began within 2 h after surgery (week 0) and continued through week 12 according to the timeline shown in **Figure 2B**.

MCC950 was administered at 10 mg/kg (intraperitoneal, once daily) and was prepared in sterile saline containing 2% dimethyl sulfoxide (DMSO). A-740003 was administered at 30 mg/kg (intraperitoneal, once daily) using the same vehicle. Control animals received vehicle injections at matched volumes and schedules. For genetic validation, Nlrp3−/− mice underwent TAC without MCC950 and were compared with WT TAC controls on the same background. Terminal functional, structural, and inflammatory outcome domains are summarized in **Figure 2C**. Detailed allocation, treatment windows, and terminal sampling procedures are provided in **Supplementary Table 1**.

### 2.5 Blood pressure monitoring and echocardiographic phenotyping

Systolic blood pressure (SBP) and heart rate were measured weekly using a noninvasive tail-cuff system (CODA, Kent Scientific, USA). Mice were acclimated to the apparatus for 3 consecutive days before baseline recording. Each session included 5 acclimation cycles followed by 15 measurement cycles, and the mean of valid cycles was used for analysis. Measurements were performed at the same time of day to reduce circadian variation.

Transthoracic echocardiography was performed weekly using a high-frequency ultrasound platform (Vevo 3100, VisualSonics, Canada) equipped with an MX550D (40 MHz) transducer. Mice were lightly anesthetized with isoflurane (1.0–1.5%) to maintain heart rate between 450 and 550 bpm. Parasternal long-axis and short-axis views were acquired. LV end-diastolic diameter, LV end-systolic diameter, posterior wall thickness, and interventricular septal thickness were measured from M-mode images. LV ejection fraction (LVEF) and fractional shortening (FS) were calculated using standard formulas.

Diastolic function was assessed by pulsed-wave Doppler across the mitral valve to derive the E/A ratio and by tissue Doppler imaging at the mitral annulus to calculate E/e′. Raw cine loops were archived for offline analysis. Representative long-axis and short-axis images were acquired under identical settings for group-level comparisons.

At terminal sampling, heart weight and lung weight were recorded, and congestion-related indices (e.g., lung weight/body weight ratio) were calculated to support heart failure phenotyping.

### 2.6 Terminal collection, euthanasia, blood processing, and tissue allocation

At prespecified terminal time points (weeks 2, 4, 8, and 12), mice were euthanized with pentobarbital sodium (150 mg/kg, intraperitoneal), followed by cervical dislocation to confirm death. Whole blood was collected by cardiac puncture into ethylenediaminetetraacetic acid (EDTA)-coated tubes. Plasma was isolated by centrifugation (1,500 × g, 15 min, 4 °C) and stored at −80 °C.

Peripheral blood mononuclear cells (PBMCs) were isolated using Ficoll-based density gradient separation (Ficoll-Paque PLUS, GE Healthcare) according to the manufacturer’s instructions and were either processed immediately or cryopreserved in 90% fetal bovine serum (FBS) and 10% DMSO.

Hearts were excised rapidly, rinsed in ice-cold PBS, and dissected to isolate the LV. LV tissue was divided into two portions: one fixed for histology/immunostaining and one snap-frozen in liquid nitrogen for RNA and protein analyses. All samples were labeled with coded identifiers to preserve blinding during downstream processing and quantification.

### 2.7 Histology, immunostaining, and quantitative image analysis

For histology, LV tissue was fixed in 4% paraformaldehyde for 24 h at room temperature, dehydrated, paraffin-embedded, and sectioned at 5 μm. General morphology was evaluated by hematoxylin and eosin (H&E) staining. Myocardial fibrosis was assessed using Masson’s trichrome and picrosirius red staining.

Collagen area fraction in the LV interstitium was quantified in ImageJ (NIH) using at least five non-overlapping fields per section and three sections per heart, sampled at predefined anatomical levels. Field selection followed a prespecified rule: fields containing large vessels or clear processing artifacts were excluded, and the remaining fields were selected using a randomized grid approach.

Cardiomyocyte cross-sectional area was measured using wheat germ agglutinin (WGA) staining. At least 100 cardiomyocytes per heart were quantified by an assessor blinded to group allocation.

To assess inflammatory infiltration and macrophage accumulation, immunohistochemistry or immunofluorescence staining was performed using antibodies against F4/80 and CD68. Nuclei were counterstained with DAPI. Images were captured using a fluorescence microscope (Zeiss Axio Observer, Germany) or confocal microscope (Leica TCS SP8, Germany) under fixed acquisition settings (constant exposure/laser power and gain within each marker batch). Quantification was performed using predefined thresholds and identical analysis macros across groups. Analysts remained blinded throughout image processing and quantification.

To improve reproducibility and clarify how each mechanistic question was operationalized, the staged mapping of specimen sources, assay platforms, and primary readouts is provided in **Supplementary Table 2**.

#### 2.7.1 Flow cytometry and immune profiling

Single-cell suspensions from LV tissue were prepared for immune phenotyping. Single-cell suspensions from LV tissue were prepared for immune phenotyping. LV tissue was minced on ice and digested in Hanks’ Balanced Salt Solution (HBSS) containing collagenase type II (1 mg/mL), dispase (1 U/mL), and DNase I (50 U/mL) at 37 °C for 30–40 min with gentle agitation. Digestion was stopped with cold complete medium, and the suspension was filtered through a 70-μm strainer. Red blood cells were lysed with ammonium-chloride-potassium (ACK) lysis buffer when required. Cells were counted, and viability was assessed by trypan blue exclusion.

Before antibody staining, cells were incubated with a fixable viability dye and Fc block (anti-CD16/32, 10 min at 4 °C). A prespecified antibody panel was used to identify leukocyte and myeloid populations, including CD45, CD11b, F4/80, Ly6C, and P2X7. For intracellular markers (e.g., iNOS and Arg1, where applicable), cells were fixed and permeabilized using a commercial kit according to the manufacturer’s protocol.

Data were acquired on a BD LSRFortessa flow cytometer (BD Biosciences, USA), with compensation established using single-stained controls. At least 100,000 live events were recorded per sample when feasible. Data analysis was performed in FlowJo (v10). The gating sequence was predefined as follows: FSC/SSC → singlets → live cells → CD45+ leukocytes → CD11b+ myeloid cells → F4/80+ macrophages. Ly6C^hi subsets were quantified within the macrophage gate. P2X7 positivity was quantified within CD11b+F4/80+ cells using fluorescence-minus-one controls to define positivity thresholds.

#### 2.7.2 Molecular assays (RT–qPCR, ELISA, immunoblotting, and caspase-1 activity)

##### 2.7.2.1 RNA extraction and RT–qPCR

Frozen LV tissue was homogenized in TRIzol reagent, and total RNA was extracted according to the manufacturer’s instructions. RNA purity was evaluated by A260/A280, and RNA integrity was verified (RIN ≥ 7.0) for qPCR panels. cDNA was synthesized using a reverse transcription kit (High-Capacity cDNA Reverse Transcription Kit, Applied Biosystems). Quantitative PCR was performed on a QuantStudio 6 system (Applied Biosystems) using SYBR Green chemistry. Cycling conditions were 95 °C for 10 min, followed by 40 cycles of 95 °C for 15 s and 60 °C for 60 s. Target genes included Nlrp3, Pycard (Asc), Casp1, Il1b, Il18, and macrophage polarization markers (Nos2 and Arg1, where applicable). GAPDH was used as the housekeeping gene. Relative expression was calculated using the 2^-ΔΔCt method. All reactions were performed in triplicate, and a prespecified Ct dispersion threshold (SD < 0.30 Ct) was applied.

##### 2.7.2.2 Plasma and tissue cytokines

IL-1β and IL-18 concentrations in plasma and LV homogenates were measured using Enzyme-Linked Immunosorbent Assay (ELISA) kits according to the manufacturers’ instructions. Samples were assayed in duplicate, and repeat testing was performed if duplicate coefficient of variation values exceeded 12%. For systemic inflammatory profiling, hsCRP and IL-6 were measured in plasma using ELISA or multiplex immunoassays. Pooled QC plasma was included on each plate to monitor batch consistency.

##### 2.7.2.3 Immunoblotting

LV tissue was lysed in ice-cold radioimmunoprecipitation assay (RIPA) buffer containing protease and phosphatase inhibitors. Protein concentration was determined by the bicinchoninic acid (BCA) assay. Equal amounts of protein (30–40 μg) were separated by dodium dodecyl sulfate-polyacrylamide gel electrophoresis (SDS-PAGE) and transferred to polyvinylidene fluoride (PVDF) membranes. Membranes were blocked in 5% nonfat milk and incubated with primary antibodies against NLRP3, ASC, caspase-1 (full-length and p20), IL-1β (pro and mature), GSDMD (full-length and cleaved GSDMD-N), and phospho-NF-κB p65/total p65, followed by horseradish peroxidase (HRP)-conjugated secondary antibodies. Bands were visualized by chemiluminescence and quantified by densitometry. β-actin or GAPDH was used as the loading control. A reference LV lysate was loaded on each gel for cross-gel normalization.

##### 2.7.2.4 Caspase-1 activity

Caspase-1 activity in cardiac immune cells or LV-derived CD45+ fractions was measured using a fluorochrome inhibitor of caspases (FLICA)-based assay and quantified by flow cytometry. Identical instrument settings were used across batches.

### 2.8 *In vitro* mechanistic validation

To test whether stress-related danger signals directly activate the NLRP3 inflammasome in immune effector cells and to distinguish upstream P2X7 blockade from direct NLRP3 inhibition, *in vitro* experiments were designed to recapitulate the sequence of ATP–P2X7 signaling, inflammasome activation, and downstream effector release. Primary macrophages were used as a validation system.

Bone marrow–derived macrophages (BMDMs) were generated from WT and Nlrp3−/− mice and differentiated for 7 days in complete Dulbecco’s Modified Eagle’s Medium (DMEM) supplemented with macrophage colony-stimulating factor (M-CSF, 20 ng/mL). Cells were plated at a uniform density and allowed to adhere overnight. Experimental groups included vehicle control, ATP stimulation, ATP plus P2X7 blockade, and ATP plus MCC950.

To model canonical two-step inflammasome activation, cells were primed with lipopolysaccharides (LPS) (100 ng/mL) for 3 h and then stimulated with ATP (5 mM, 30 min). For inhibitor conditions, A-740003 (10 μM) or MCC950 (10 μM) was added 30 min before ATP stimulation and maintained during the activation period. Cell lysates and supernatants were collected at prespecified time points.

Primary readouts included ASC speck formation (immunofluorescence/confocal microscopy), caspase-1 activation (FLICA assay and/or cleaved caspase-1 p20 immunoblotting), cytokine maturation and release (IL-1β and IL-18 by ELISA), and cell injury (lactate dehydrogenase release). P2X7 engagement and blockade efficacy were verified using YO-PRO-1 uptake and/or surface P2X7 expression by flow cytometry. Each condition was repeated in at least three independent biological replicates.

### 2.9 Human translational validation and cohort governance

To evaluate the clinical translatability of the inflammasome-axis findings, a prospective observational cohort was established in adults with hypertension-related chronic heart failure. The study was approved by the Institutional Review Board of Xinhua Hospital, affiliated with Dalian University (approval no. IRB-2025-147-01), and it was conducted in accordance with the Declaration of Helsinki. Written informed consent was obtained before any study-specific procedures.

Participants were enrolled during a clinically stable period, with exclusion of acute decompensation, active infection, or recent major clinical events that could substantially alter inflammatory profiles. At baseline, demographic and clinical characteristics, medication use, and echocardiographic indices were recorded using standardized case report forms. Peripheral blood was collected into EDTA tubes for plasma and peripheral blood mononuclear cell (PBMC) processing. Plasma was separated within 2 h, aliquoted, screened for hemolysis, and stored at −80 °C. PBMCs were isolated by Ficoll density gradient centrifugation, assessed for viability, and cryopreserved using standardized procedures to reduce pre-analytical variability.

Follow-up sampling was performed within a prespecified window (12 ± 2 weeks) to assess the temporal stability of pathway markers and their associations with heart failure severity and cardiac function. Plasma assays included IL-1β, IL-18, IL-6, and hsCRP (ELISA or multiplex assays), and extracellular ATP was measured by a luciferase-based assay where applicable. PBMC pathway activation was assessed by RT–qPCR of core inflammasome genes (NLRP3, PYCARD, CASP1, IL1B, and IL18). In a prespecified subset, flow cytometry was used to quantify P2X7 expression in circulating myeloid cells under a predefined gating strategy.

All clinical data and biospecimens were managed using de-identified study IDs. Access to the dataset was role-restricted, and audit trails were maintained for data export and analysis. Standard operating procedures governed sample handling, aliquoting, storage, and batch processing. Detailed inclusion/exclusion criteria and human-cohort quality-control procedures are provided in **Supplementary Table 3A**. Animal randomization, blinding, and assay quality-control procedures are summarized separately in **Supplementary Table 3B**.

### 2.10 Statistical analysis

Continuous variables were assessed for distributional characteristics before analysis. Normally distributed variables are presented as mean ± standard deviation and were compared using Student’s t-test (two groups) or one-way analysis of variance (ANOVA) (multiple groups) with appropriate *post hoc* correction. Non-normally distributed variables are presented as median (interquartile range) and were compared using the Mann–Whitney U test (two groups) or Kruskal–Wallis test (multiple groups). Categorical variables are reported as counts (percentages) and were compared using the χ² test or Fisher’s exact test, as appropriate.

Longitudinal blood pressure and echocardiographic measurements were analyzed using linear mixed-effects models, with group, time, and group-by-time interaction as fixed effects and animal identity as a random effect. Baseline values were included as covariates where applicable. Prespecified pairwise comparisons were adjusted for multiple testing.

Terminal histologic, flow cytometry, and molecular endpoints were analyzed using multivariable regression models to examine associations between inflammasome-axis markers and remodeling or functional outcomes, with adjustment for prespecified covariates. For analyses addressing the proposed mechanistic chain, mediation and path models were used to estimate indirect effects linking ATP–P2X7 signaling, NLRP3 activation, and remodeling phenotypes.

Missing data were handled using prespecified rules. Sensitivity analyses were performed using complete-case datasets and alternative assumptions regarding missingness. All tests were two-sided, and P < 0.05 was considered statistically significant. Analyses were performed using R (v4.3.3) and GraphPad Prism (v10). Mixed-effects models were implemented using standard R packages.

## 3 Results

### 3.1 TAC model validation, stage classification, and intervention-related stage redistribution

**Figure 3:**
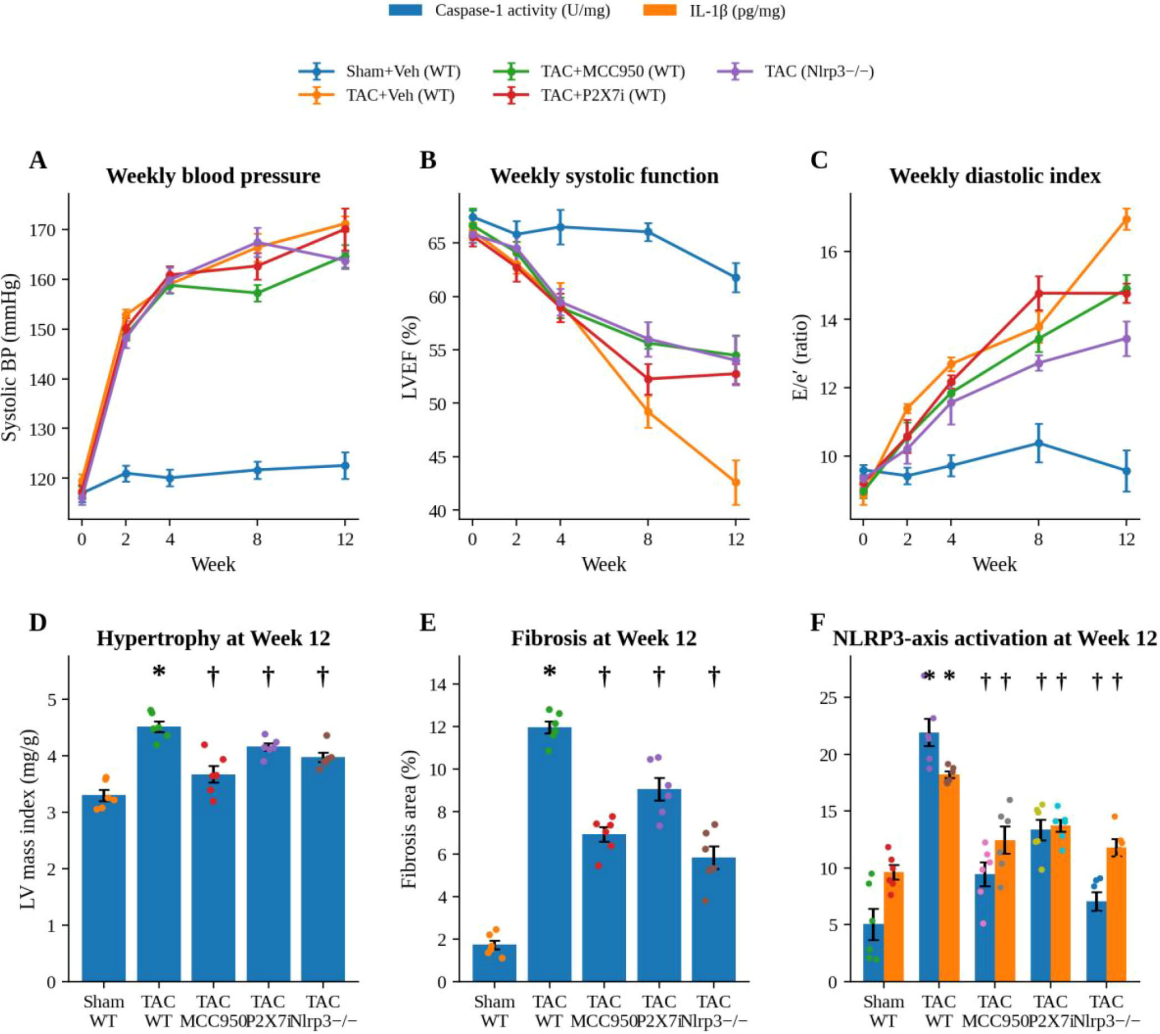
Validation of transverse aortic constriction (TAC)-induced pressure overload and progressive cardiac remodeling. **(A)** Weekly systolic blood pressure measured by tail cuff. **(B)** Longitudinal change in left ventricular (LV) systolic function [LV ejection fraction (LVEF)]. **(C)** Time-dependent change in diastolic load assessed by E/e′. **(D)** LV hypertrophy at week 12 (LV mass index). **(E)** Myocardial fibrosis at week 12. **(F)** Representative inflammasome-axis activation readouts at week 12, including caspase-1 activity and IL-1β. Data are shown according to the prespecified grouping and sampling framework. Statistical comparisons were performed as described in the Methods. *P < 0.05 and **P < 0.01 vs Sham+Vehicle (WT); †P < 0.05 and ††P < 0.01 vs TAC+Vehicle (WT), unless otherwise indicated.

We first confirmed that TAC produced sustained pressure overload and progressive cardiac remodeling consistent with hypertension-related chronic heart failure (CHF). Weekly tail-cuff measurements showed a persistent increase in systolic blood pressure after TAC, indicating stable afterload stress throughout follow-up (**Figure 3A**). Serial echocardiography further demonstrated progressive deterioration in cardiac function, including a time-dependent decline in LVEF (**Figure 3B**) and worsening diastolic load as reflected by E/e′ (**Figure 3C**). By week 12, TAC mice also showed increased LV hypertrophy indices (**Figure 3D**), marked expansion of myocardial fibrosis (**Figure 3E**), and elevation of inflammasome-related readouts (**Figure 3F**), together supporting successful establishment of a pressure overload–driven CHF phenotype.

Preoperative comparisons at week 0 showed no meaningful between-group differences in age, body weight, blood pressure, or baseline echocardiographic parameters, supporting adequate baseline comparability. In addition, TAC model quality metrics were acceptable, with a banding success rate >92% and perioperative mortality <8% across TAC groups. Detailed baseline comparability and model-validation metrics are provided in **Supplementary Table 4**.

To support stage-based mechanistic analyses, animals were classified at week 12 into three prespecified categories using integrated functional, structural, and congestion indicators: compensated hypertrophy, transition (early CHF), and established CHF. TAC+Vehicle animals were predominantly classified as established CHF, whereas both MCC950-treated mice and Nlrp3−/− TAC mice showed redistribution toward earlier stages, with greater preservation of LVEF and lower remodeling and inflammatory burden. The P2X7 inhibitor group showed an intermediate pattern, consistent with partial upstream pathway suppression. This stage framework was then used to examine whether inflammasome activation scales with disease severity and whether ATP–P2X7 signaling precedes NLRP3 inflammasome execution.

### 3.2 Stage-dependent escalation of inflammasome signaling across cardiac function, remodeling, and biomarker domains

After establishing the TAC stage framework, we examined whether ATP–P2X7–NLRP3 signaling increased in a stage-dependent manner and whether this increase tracked CHF severity across prespecified parameter domains.

#### 3.2.1 Cardiac function domain

Inflammasome-axis activation was closely associated with worsening functional indices at week 12. In multivariable models, higher caspase-1 activity, IL-1β, and GSDMD cleavage were independently associated with lower LVEF, whereas higher caspase-1 and IL-18 levels were associated with worse diastolic loading (higher E/e′). In addition, inflammasome-related markers were positively associated with NT-proBNP and congestion-related indices, indicating that stronger inflammasome activation coincided with more advanced functional decompensation. Full model coefficients and confidence intervals are provided in **Supplementary Table 5A**.

These functional associations were consistent with the staged phenotype. Animals in the established CHF category showed the highest burden of inflammasome-related signaling and the most severe impairment in systolic and diastolic function, while compensated animals showed lower effector activation and relatively preserved cardiac performance.

#### 3.2.2 Cardiac remodeling domain

Inflammasome signaling also tracked structural remodeling severity. Higher inflammasome-related effectors were independently associated with greater LV mass index, increased myocardial fibrosis, and enhanced macrophage accumulation. Notably, IL-1β and ASC speck burden showed strong associations with fibrosis, supporting a link between inflammasome activation and extracellular matrix remodeling. These findings indicate that inflammasome activation is not only a correlate of functional decline but is also tightly linked to the structural remodeling process itself (**Supplementary Table 5A**).

When disease stage was modeled as an ordinal outcome, caspase-1 activity, IL-1β, and GSDMD cleavage remained associated with a higher probability of advanced stage membership, even when systolic blood pressure was considered in the model. This supports the interpretation that inflammasome signaling contributes explanatory value beyond pressure load alone (**Supplementary Table 5B**).

#### 3.2.3 Signaling biomarker and causal-chain domain

Across disease stages, the ATP–P2X7–NLRP3 axis showed a stepwise biological pattern consistent with progressive transition from upstream danger signaling to inflammasome assembly and downstream effector activation. In compensated hypertrophy, upstream pathway engagement was detectable, but downstream effector injury signals were comparatively limited. During the transition stage, ATP/P2X7-related activity increased together with stronger NLRP3–ASC–caspase-1 activation and greater cytokine maturation. In established CHF, sustained inflammasome amplification coincided with pronounced immune infiltration, fibrosis expansion, and worsening cardiac performance.

**Figure 4:**
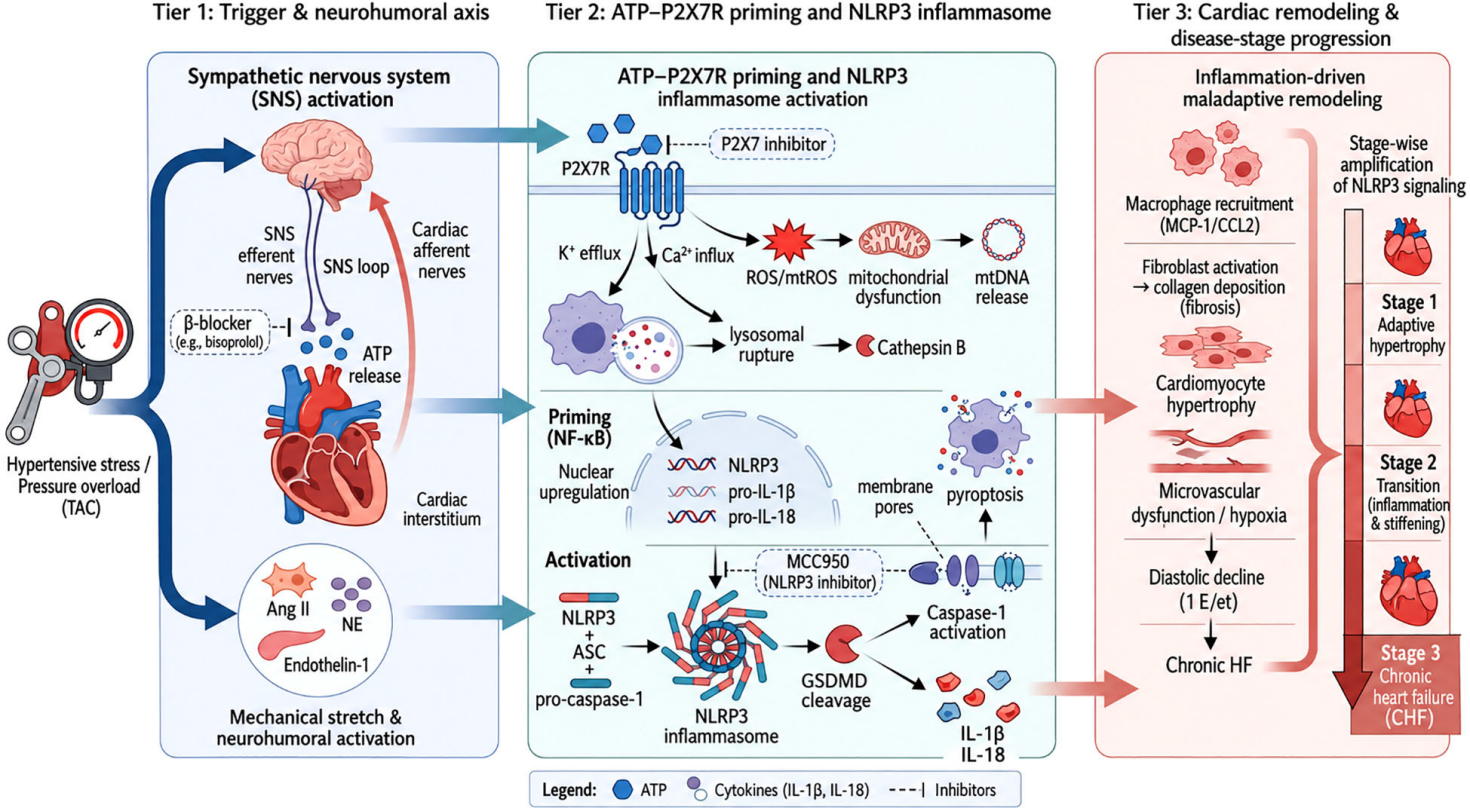
Stage-informed mechanistic framework linking pressure overload to ATP–P2X7–NLRP3 signaling and chronic heart failure (CHF) progression. Schematic overview of the proposed sequence from pressure overload to extracellular ATP accumulation and P2X7 activation, followed by NLRP3 inflammasome assembly, caspase-1 activation, IL-1β/IL-18 maturation, pyroptosis-related effector signaling, and downstream inflammatory-fibrotic remodeling that contributes to cardiac dysfunction. Pharmacologic (P2X7 inhibitor, MCC950) and genetic (Nlrp3−/−) interventions are mapped onto the inferred pathway hierarchy.

To summarize this staged sequence, the mechanistic framework linking pressure overload to ATP–P2X7 signaling, inflammasome activation, pyroptosis-related effector signaling, and maladaptive remodeling is illustrated in **Figure 4**. This framework was then tested statistically. Mediation and path analyses supported a sequential structure in which ATP–P2X7 signaling aligned upstream of caspase-1 and IL-1β, with fibrosis acting as a partial mediator linking inflammasome effectors to functional decline (**Supplementary Table 5C**).

Finally, intervention-anchored contrasts provided causal support for this hierarchy. Pharmacologic NLRP3 inhibition (MCC950) and genetic Nlrp3 deletion produced larger reductions in inflammasome activity and remodeling indices, with greater preservation of cardiac function, whereas P2X7 inhibition yielded a partial but consistent benefit profile. These contrasts support the interpretation that ATP–P2X7 acts upstream, while NLRP3-centered signaling is a stronger proximal driver of downstream remodeling and dysfunction (**Supplementary Table 5D**).

Together, these results support the next analysis step: direct testing of whether ATP–P2X7 signaling functions as the upstream gating mechanism that couples pressure overload stress to NLRP3 inflammasome activation.

### 3.3 Evidence that extracellular ATP–P2X7 signaling couples pressure overload to NLRP3 inflammasome activation

**Figure 5:**
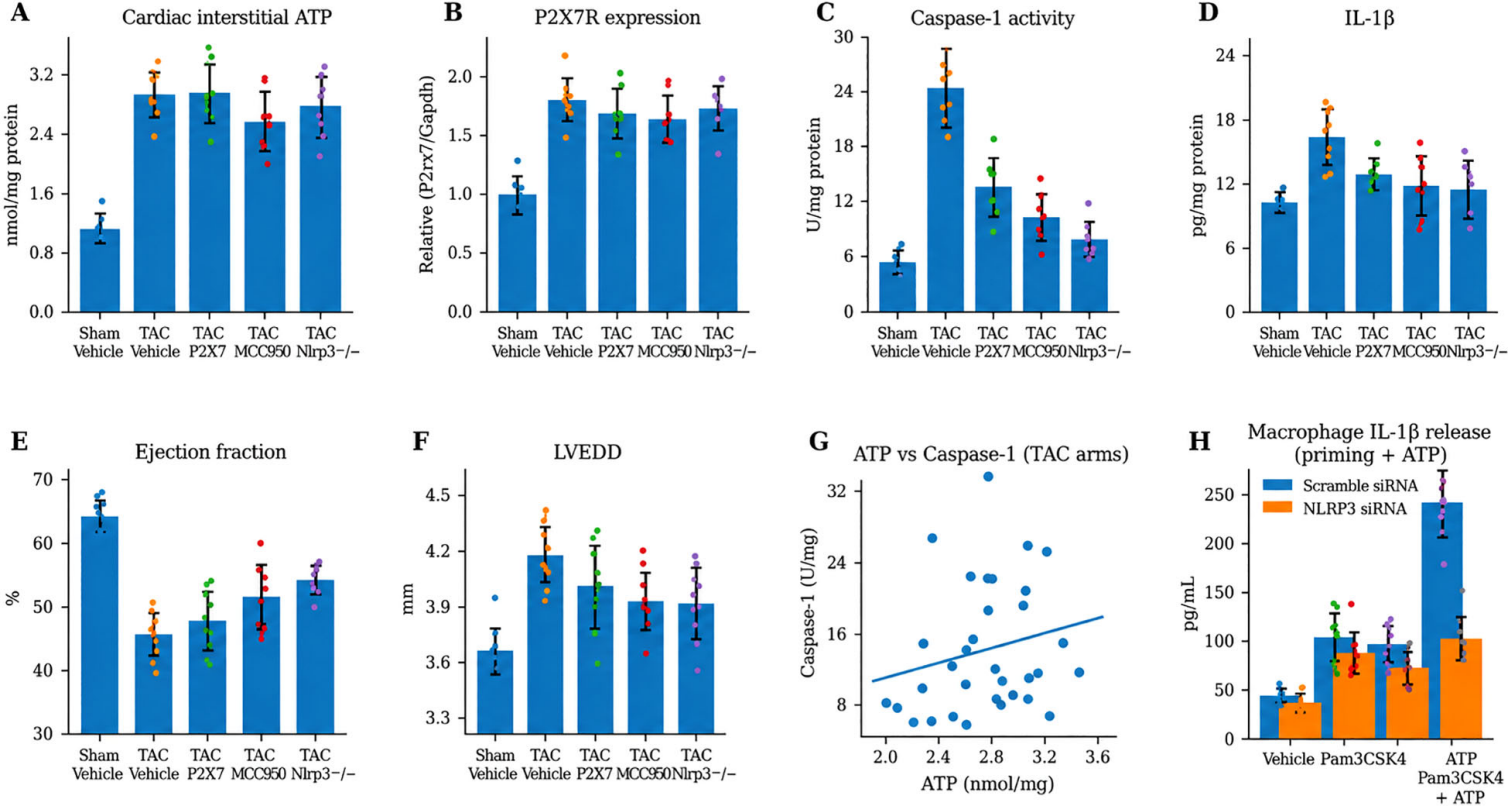
Extracellular ATP–P2X7 signaling is associated with NLRP3 inflammasome activation and cardiac dysfunction under transverse aortic constriction (TAC). **(A)** Myocardial extracellular ATP levels. **(B)** P2X7 receptor expression/activity readout. **(C)** Caspase-1 activity. **(D)** Mature IL-1β levels. **(E)** Left ventricular ejection fraction (LVEF). **(F)** Left ventricular end-diastolic diameter (LVEDD). **(G)** Association between myocardial extracellular ATP and caspase-1 activity in TAC animals. **(H)** *In vitro* IL-1β release from primed macrophages after ATP stimulation with or without NLRP3 disruption. Statistical analyses were performed as described in the Methods.

We next examined whether extracellular ATP–P2X7 signaling tracked with inflammasome activation and cardiac deterioration under TAC and across mechanistic interventions. TAC markedly increased myocardial extracellular ATP levels (**Figure 5A**) and was accompanied by higher P2X7 receptor expression/activity indices (**Figure 5B**). In parallel, inflammasome activation was enhanced, as reflected by increased caspase-1 activity (**Figure 5C**) and greater mature IL-1β release (**Figure 5D**). These molecular changes coincided with reduced systolic function (**Figure 5E**) and ventricular dilation (**Figure 5F**), supporting a link between upstream danger signaling and progressive cardiac dysfunction.

Across TAC animals, myocardial extracellular ATP levels were positively associated with caspase-1 activity (**Figure 5G**), consistent with an upstream relationship between danger-signal accumulation and inflammasome execution. Intervention comparisons further clarified the pathway hierarchy. Pharmacologic P2X7 inhibition attenuated inflammasome-related readouts, whereas direct NLRP3 inhibition (MCC950) or genetic Nlrp3 deletion produced broader suppression of downstream inflammatory effectors together with stronger preservation of cardiac structure and function.

Complementary *in vitro* experiments supported this staged pathway interpretation. In primed macrophages, ATP stimulation increased IL-1β release, and this response was substantially reduced by NLRP3 disruption (**Figure 5H**). Taken together, the *in vivo* and *in vitro* findings support a model in which extracellular ATP–P2X7 signaling functions as an upstream gate linking pressure-overload stress to NLRP3 inflammasome activation.

### 3.4 Upstream pathway modulation attenuates inflammasome activation and remodeling under maintained pressure overload

**Figure 6:**
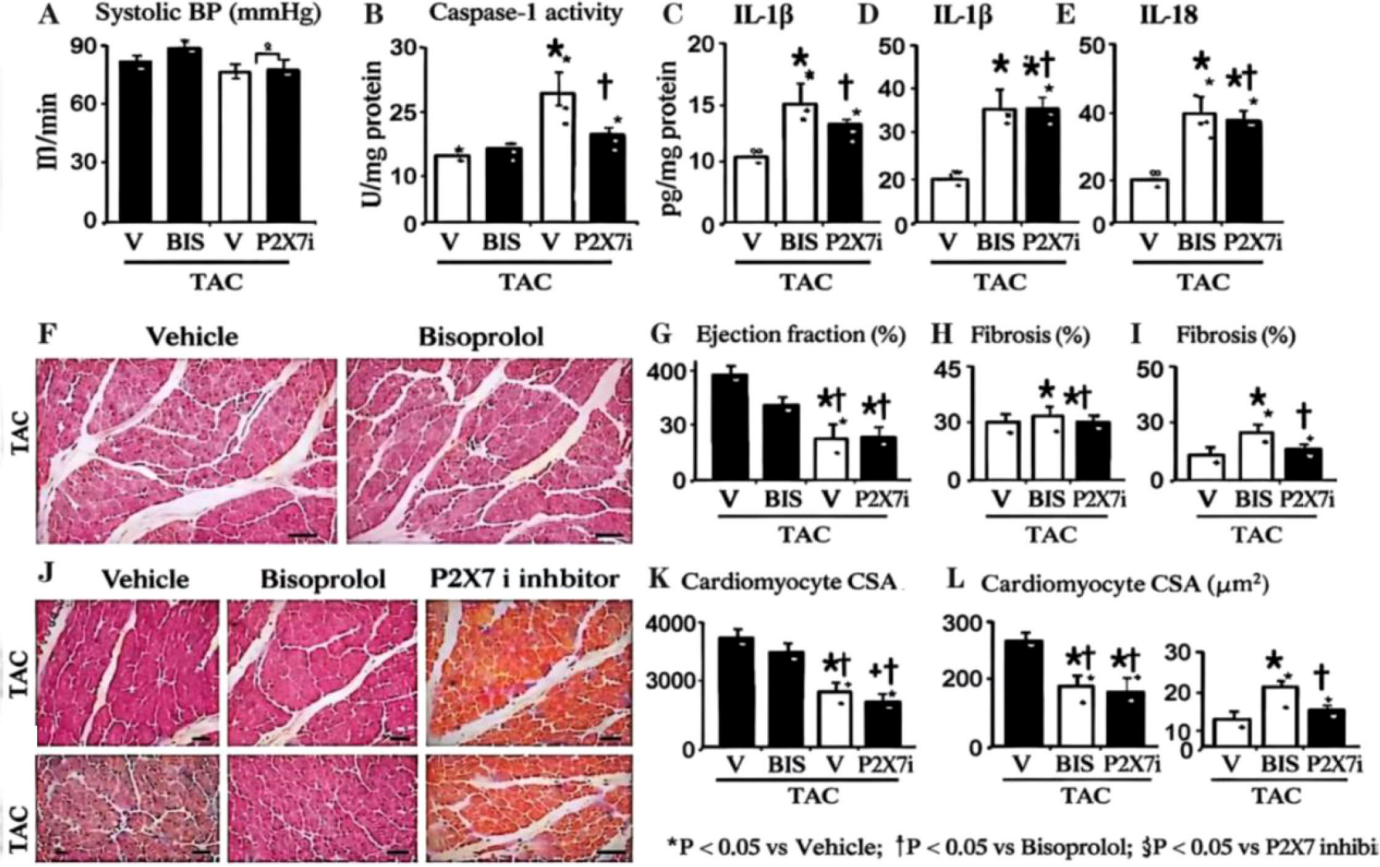
Upstream pathway modulation attenuates inflammasome signaling and cardiac remodeling under maintained pressure overload. **(A)** Systolic blood pressure across transverse aortic constriction (TAC) groups under the indicated intervention conditions. **(B)** Left ventricular caspase-1 activity. **(C, D)** IL-1β readouts. **(E)** IL-18 levels. **(F, J)** Representative myocardial histology across indicated groups. **(G)** Ejection fraction. **(H, I)** Quantification of myocardial fibrosis. **(K, L)** Cardiomyocyte cross-sectional area (CSA) measurements. All quantifications followed the prespecified field-selection and blinding procedures described in the Methods. *P < 0.05 and **P < 0.01 vs TAC+Vehicle; †P < 0.05 vs TAC+Bisoprolol; §P < 0.05 vs TAC+P2X7 inhibitor, unless otherwise indicated.

We next tested whether interrupting upstream danger-signal entry could reduce downstream inflammasome activation and cardiac remodeling while preserving the pressure-overload condition. Across TAC intervention groups, systolic blood pressure remained within the pressure-overload range (**Figure 6A**), indicating that the observed downstream differences were not attributable to loss of the TAC stimulus.

At the molecular level, TAC increased inflammasome effector signaling, including elevated caspase-1 activity (**Figure 6B**), increased IL-1β readouts (**Figures 6C, D**), and higher IL-18 levels (**Figure 6E**). Upstream pathway modulation was associated with attenuation of these readouts, indicating reduced inflammasome execution despite persistent afterload stress.

These molecular changes were accompanied by improvements in organ-level remodeling and cardiac performance. Histological comparisons showed group-level differences in myocardial architecture (**Figures 6F, J**), and echocardiography demonstrated better-preserved ejection fraction under intervention conditions than in TAC controls (**Figure 6G**). Fibrosis burden was lower in intervention groups (**Figures 6H, I**), and cardiomyocyte hypertrophy was reduced, as indicated by smaller cardiomyocyte cross-sectional area (**Figures 6K, L**). Collectively, these data show coordinated improvement across inflammasome signaling, structural remodeling, and cardiac function under upstream pathway modulation.

### 3.5 Pharmacologic NLRP3 inhibition preserves cardiac structure and function during pressure overload–induced remodeling

**Figure 7:**
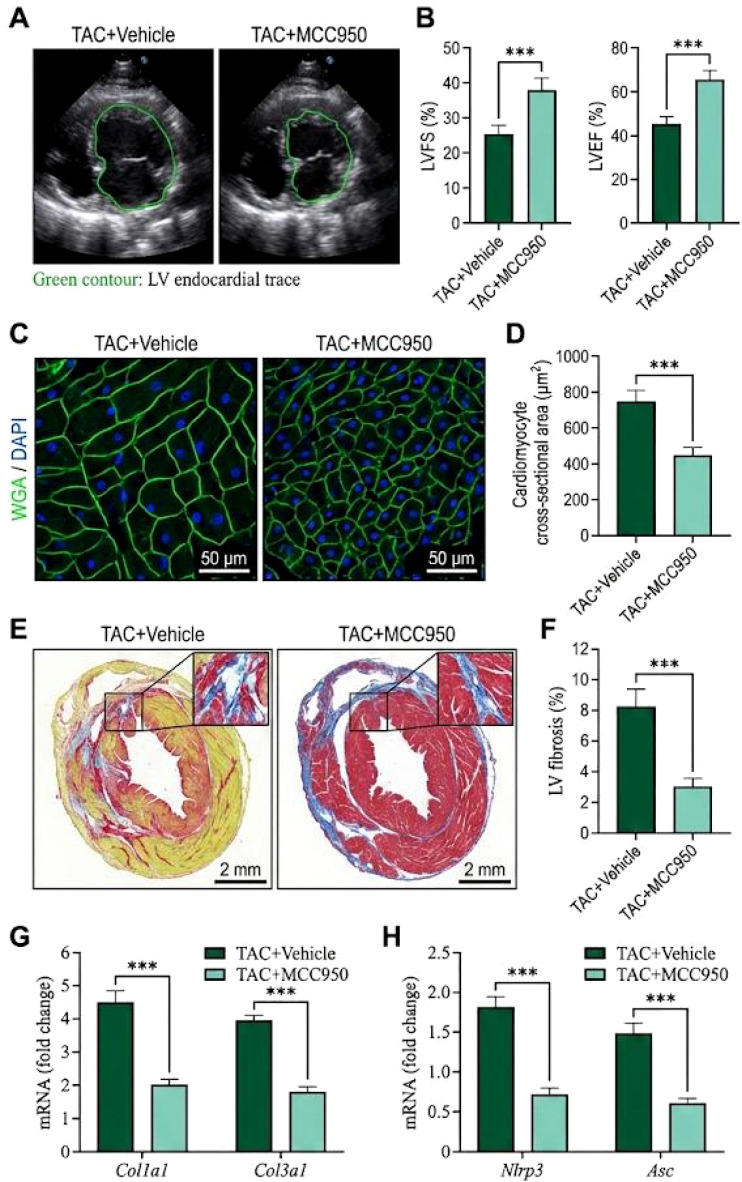
MCC950 preserves cardiac function and attenuates remodeling under transverse aortic constriction (TAC). **(A)** Representative echocardiographic images from TAC+Vehicle and TAC+MCC950 groups. **(B)** Quantification of systolic function parameters. **(C)** Representative staining images showing cardiomyocyte morphology. **(D)** Cardiomyocyte cross-sectional area. **(E)** Representative histologic images of myocardial fibrosis. **(F)** Quantification of fibrosis burden. **(G)** mRNA expression of fibrosis-related genes. **(H)** mRNA expression of inflammasome-pathway genes. Statistical analyses were performed as described in the Methods. ***P < 0.001 vs TAC+Vehicle.

We next focused on the effect of pharmacologic NLRP3 inhibition on cardiac structure and function under TAC. Compared with TAC+Vehicle animals, MCC950-treated mice showed better-preserved systolic performance (**Figures 7A, B**).

Structural remodeling was also attenuated. Histologic and immunostaining analyses showed smaller cardiomyocyte profiles in the MCC950 group (**Figures 7C, D**), together with reduced myocardial fibrosis (**Figures 7E, F**). At the molecular level, MCC950 treatment was associated with lower expression of fibrosis-related transcripts and reduced expression of inflammasome-pathway genes (**Figures 7G, H**). These findings are consistent with the hypothesis that NLRP3 inhibition mitigates both inflammatory signaling and downstream remodeling under sustained pressure overload.

### 3.6 NLRP3 inhibition reduces myocardial inflammatory burden and inflammasome effector signaling

**Figure 8:**
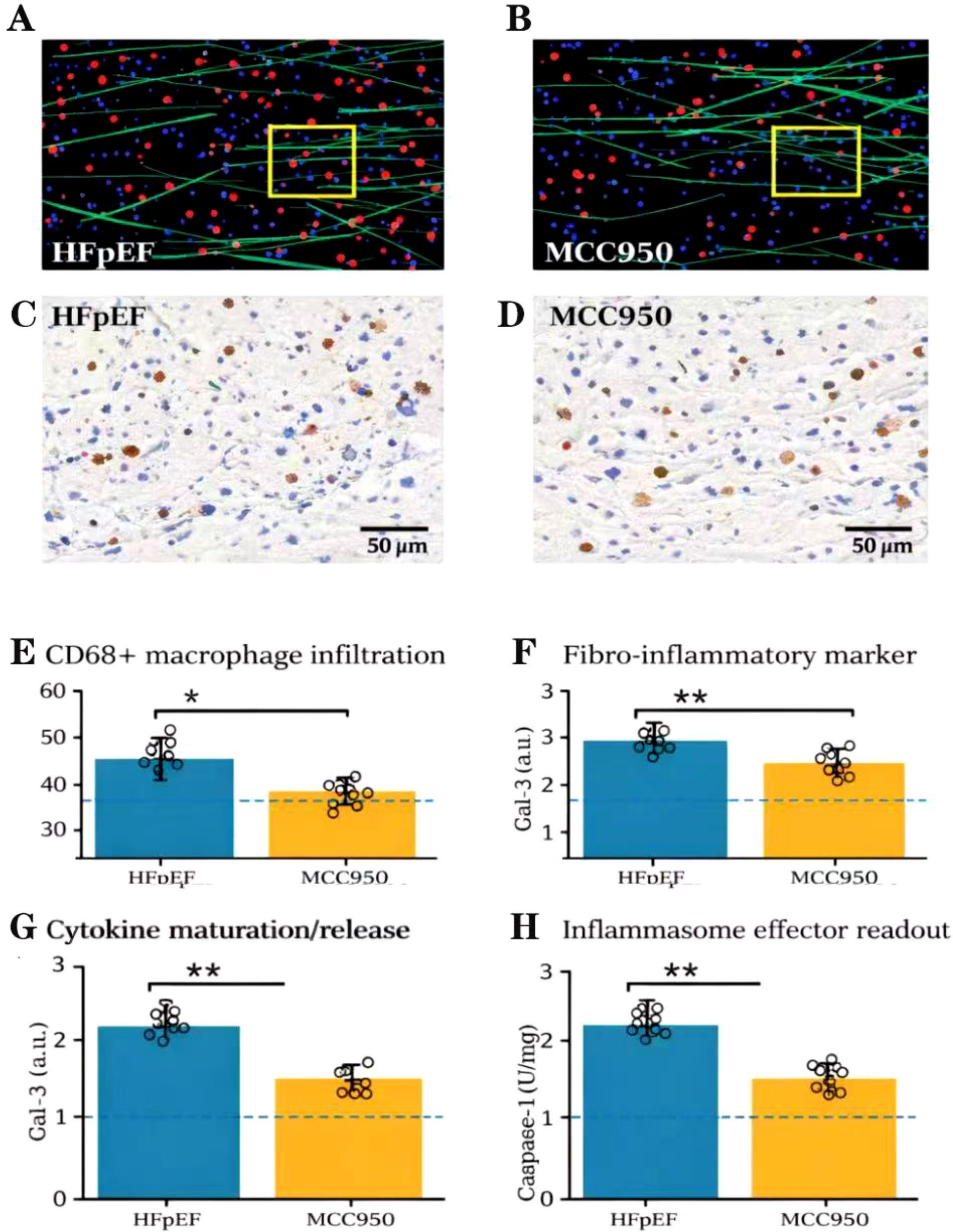
MCC950 reduces myocardial inflammatory burden and inflammasome effector signaling in the left ventricle. **(A, B)** Representative myocardial histology in transverse aortic constriction TAC+Vehicle and TAC+MCC950 groups (boxed regions shown at higher magnification). **(C, D)** Representative immunostaining images (scale bar, 50 μm). **(E)** Quantification of CD68+ macrophage infiltration. **(F)** Quantification of fibro-inflammatory marker levels (as labeled). **(G)** Cytokine maturation/release-related readout (as labeled). **(H)** Caspase-1 activity (U/mg). Statistical analyses were performed as described in the Methods. *P < 0.05 and **P < 0.01 vs TAC+Vehicle.

As shown by the stage-based and multivariable analyses (**Supplementary Tables S4**, **S5**), macrophage accumulation and inflammasome effector signaling increased with TAC progression. We therefore next assessed whether NLRP3 inhibition reduced myocardial inflammatory burden. TAC+Vehicle hearts showed prominent macrophage infiltration together with elevated inflammasome activity. Representative histology and immunostaining images are shown in **Figures 8A–D**.

MCC950 treatment was associated with lower CD68+ macrophage density (**Figure 8E**) and reduced fibro-inflammatory marker levels (**Figure 8F**). At the effector level, MCC950 also reduced cytokine maturation/release-related signals (**Figure 8G**) and caspase-1 activity (**Figure 8H**). These changes were directionally consistent across cellular and molecular readouts, supporting a coordinated reduction in inflammatory burden and inflammasome execution in the left ventricle under NLRP3 inhibition.

Taken together, the results from Sections 3.3–3.6 support a hierarchical mechanism in which extracellular ATP–P2X7 signaling acts upstream of NLRP3 inflammasome activation, while direct NLRP3 inhibition provides stronger suppression of downstream inflammatory remodeling and greater preservation of cardiac structure and function.

## 4 Discussion

Under sustained pressure overload, the transition from compensated hypertrophy to overt heart failure is unlikely to be fully explained by mechanical burden alone. Our observations suggest that an early inflammatory shift, together with the establishment of self-reinforcing immune-remodeling interactions, appears to represent a critical inflection point in disease evolution (15). In the present study, enhanced inflammasome effector activity and increased maturation of pro-inflammatory cytokines emerged early after pressure loading and preceded progressive immune-cell recruitment, interstitial fibrosis, and structural deterioration, ultimately contributing to persistent diastolic impairment and reduced functional reserve (16). The temporal sequence captured across disease stages is therefore consistent with an inflammation-initiated trajectory, offering a mechanistic perspective on why individuals exposed to comparable hemodynamic stress may exhibit markedly different remodeling dynamics and therapeutic responsiveness (17).

A principal mechanistic contribution of our work is the identification of extracellular ATP–P2X7 signaling as an upstream gate linking pressure stress to activation of the NLRP3 inflammasome (18). In our model, elevations in extracellular ATP and heightened P2X7 engagement paralleled inflammasome priming and effector activation, supporting a cascade in which danger signals may lower the threshold for sustained inflammatory output (19). Given that targeted interference with this pathway substantially altered downstream responses, the ATP–P2X7 connection may be interpreted as a functionally relevant regulatory step rather than a simple association (20).

At the tissue level, the coordinated decline in inflammasome readouts and inflammatory-cell infiltration observed after intervention suggests that the system behaves as an interruptible amplification circuit (21). NLRP3 activation drives caspase-1–dependent cytokine maturation and membrane permeabilization processes, thereby intensifying paracrine injury signaling and expanding inflammatory recruitment (22, 23). Within the framework supported by our data, this environment favors macrophage accumulation and reinforcement of pro-fibrotic programs, accelerating extracellular matrix deposition and progressively impairing ventricular compliance. The consequent elevation in filling pressures provides a plausible mechanistic bridge to the diastolic dysfunction documented during progression, even in situations where primary load control appears adequate (24).

The intervention experiments further strengthen the inference of causality. Pharmacologic inhibition of NLRP3 produced parallel reductions in inflammatory activity and adverse remodeling, accompanied by measurable improvement in cardiac performance (25). MCC950 treatment limited hypertrophy and fibrosis, reduced macrophage infiltration, and attenuated pro-fibrotic and inflammatory signaling, closely mirroring the staged cascade identified in our analyses (26). Together with evidence linking inflammasome activation to mitochondrial stress amplification (27), these findings are compatible with a model in which ATP–P2X7 signaling initiates inflammatory engagement while metabolic injury helps sustain it across disease stages (28).

From a translational perspective, this framework highlights the potential value of stage-aware intervention. Targeting upstream triggers may mitigate initiation intensity, whereas blocking downstream execution could restrict propagation, thereby suggesting a strategy for identifying inflammation-dominant phenotypes and refining therapeutic timing in hypertension-related heart failure. Importantly, when interpreted alongside the limitations inherent to experimental pressure-overload models, these findings provide a structured foundation for future studies aimed at resolving cell-specific contributions, improving the temporal precision of intervention, and facilitating translation toward clinically heterogeneous HFpEF populations.

## 5 Limitations

Despite the mechanistic coherence of the findings, several limitations should be acknowledged. First, although the TAC model reliably reproduces pressure overload–induced remodeling, it represents an accelerated and relatively uniform injury paradigm that may not fully capture the heterogeneity, comorbidity burden, and long-term trajectories observed in clinical heart failure populations. Second, while pharmacologic inhibition and genetic deletion consistently attenuated inflammasome activation, off-target or compensatory immune mechanisms cannot be completely excluded, particularly within complex multicellular cardiac niches. Third, our analyses focused primarily on ventricular tissue–level responses; cell-type-specific contributions, including those from endothelial, fibroblast, or resident immune subsets, warrant higher-resolution approaches in future investigations. Fourth, although functional and structural improvements were evident during the observation window, longer follow-up will be necessary to determine whether interruption of the ATP–P2X7–NLRP3 axis confers durable protection against late-stage decompensation.

These considerations define important directions for subsequent translational and mechanistic studies while not diminishing the central conclusion that inflammasome engagement operates as a modifiable driver of pressure overload–associated cardiac deterioration.

## 6 Conclusion

This study demonstrates that chronic pressure overload engages extracellular ATP–P2X7 signaling as an upstream initiating mechanism that sustains NLRP3 inflammasome activation and perpetuates inflammatory amplification. By resolving this pathway across disease stages, our findings link mechanical stress to innate immune activation and clarify how sterile inflammation contributes to progressive hypertensive remodeling. In line with this framework, persistent pathway activity was associated with macrophage accumulation, advancing fibrosis, structural deterioration, and worsening cardiac performance. Importantly, both pharmacologic inhibition and genetic disruption interrupted this feed-forward inflammatory circuit, yielding coordinated improvements in myocardial structure and function. Taken together, these results position the ATP–P2X7–NLRP3 axis as a causally relevant driver rather than a passive marker of disease severity, and support stage-aware, mechanism-guided intervention strategies that target upstream inflammatory initiation in hypertension-related heart failure.

## Data Availability

The raw data supporting the conclusions of this article will be made available by the authors, without undue reservation.
